# Effect of heart rate on left ventricular longitudinal myocardial function in type 2 diabetes mellitus

**DOI:** 10.1186/s12933-021-01278-7

**Published:** 2021-04-24

**Authors:** Yuki Yamauchi, Hidekazu Tanaka, Shun Yokota, Yasuhide Mochizuki, Yuko Yoshigai, Hiroaki Shiraki, Kentaro Yamashita, Yusuke Tanaka, Ayu Shono, Makiko Suzuki, Keiko Sumimoto, Kensuke Matsumoto, Yushi Hirota, Wataru Ogawa, Ken-ichi Hirata

**Affiliations:** 1 Division of Cardiovascular Medicine, Department of Internal Medicine, Kobe University Graduate School of Medicine, 7-5-2, Kusunoki-cho, Chuo-ku, Kobe, 650-0017 Japan; 2 Division of Diabetes and Endocrinology, Department of Internal Medicine, Kobe University Graduate School of Medicine, Kobe, Japan

**Keywords:** Type 2 diabetes mellitus, Heart rate, Global longitudinal strain, Echocardiography

## Abstract

**Background:**

Left ventricular (LV) longitudinal myocardial dysfunction is considered a marker of preclinical LV dysfunction in patients with type 2 diabetes mellitus (T2DM). High heart rate (HR) is associated with cardiovascular outcomes, but the effect of HR on LV longitudinal myocardial function in T2DM patients is uncertain.

**Methods:**

We studied 192 T2DM patients with preserved LV ejection fraction (LVEF), and 81 age-, sex-, and LVEF-matched healthy volunteers. HR was measured as the average HR during echocardiography, and high HR was defined as resting HR ≥ 70 beats/minute. LV longitudinal myocardial function was assessed as global longitudinal strain (GLS). The predefined cutoff for subclinical LV dysfunction was set at GLS < 18%.

**Results:**

GLS in T2DM patients with high HR was significantly lower than that in T2DM patients with low HR (16.3% ± 4.2% vs. 17.8% ± 2.8%; P = 0.03), whereas GLS in normal subjects with high and low HR was similar (20.3 ± 1.7% vs. 20.3 ± 2.0%; P = 0.99). Multivariable logistic regression analysis showed that high HR (odds ratio: 1.04; 95% confidence interval: 1.01–1.07; P = 0.01) was independently associated with GLS < 18% in T2DM patients as well as HbA1c, T2DM duration, LVEF, body mass index, and mitral inflow E and mitral e’ annular velocity ratio. One sequential logistic model evaluating the associations between GLS < 18% and clinical variables in T2DM patients showed an improvement with the addition of LVEF and E/e’ (P < 0.001) and a further improvement with the addition of high HR (P < 0.001).

**Conclusion:**

Compared with normal subjects, resting HR was associated with LV longitudinal myocardial function in asymptomatic T2DM patients with preserved LVEF. Our findings provide new insights on the management of T2DM patients.

**Supplementary Information:**

The online version contains supplementary material available at 10.1186/s12933-021-01278-7.

## Background

Heart failure (HF) with preserved ejection fraction (HFpEF) currently accounts for approximately half of all HF cases, and its prevalence relative to that of HF with reduced ejection fraction (HFrEF) continues to rise at an alarming rate of 1% per year. Stage A HF is crucially important for the development of HFpEF and requires management with the long-term goal of avoiding HF progression. Stage B HF patients are ideal targets for HF prevention. Type 2 diabetes mellitus (T2DM) is another well-known risk factor for HF and is an important comorbid disease of Stage A HF, similar to hypertension. Left ventricular (LV) longitudinal myocardial dysfunction, assessed in terms of low global longitudinal strain (GLS), is a sensitive marker for early subtle abnormalities in LV myocardial performance, helpful for predicting outcomes for various cardiac diseases, and superior to conventional echocardiographic indices such as LV ejection fraction (LVEF) and mitral inflow E and mitral e’ annular velocity ratio (E/e’) [1–5]. In addition, LV longitudinal myocardial dysfunction is altered in Stage A HF patients and can be an early marker of LV dysfunction, which in turn indicates cardiovascular morbidity and mortality. Thus, LV longitudinal myocardial dysfunction is also considered a sensitive marker of a preclinical form of LV dysfunction in patients with T2DM and preserved LVEF without overt HF [6–12]. Therefore, LV longitudinal myocardial dysfunction should be considered the first marker of a preclinical form of T2DM-related cardiac dysfunction, known as diabetic cardiomyopathy.

High resting heart rate (HR) is a known marker of cardiovascular outcomes for various categories of HF patients, especially HFrEF patients, and HR-lowering therapy has a positive impact on outcomes in HFrEF patients [13]. However, the effect of resting HR on LV longitudinal myocardial function in T2DM patients is unclear. Therefore, this study aimed to investigate the association of resting HR with LV longitudinal myocardial function in asymptomatic T2DM patients with preserved LVEF without coronary artery disease.

## Methods

### Study population

In this study, a total of 192 asymptomatic T2DM patients with preserved LVEF who were admitted to Kobe University Hospital between June 2013 and March 2020 were retrospectively enrolled. The preliminary exclusion criteria were as follows: (1) history of coronary artery disease, (2) LVEF < 50%, (3) previous history of open-heart surgery or congenital heart disease, (4) severe renal dysfunction defined as a glomerular filtration rate < 30 mL/min/1.73 m^2^, (5) uncontrolled hypertension with blood pressure > 180/100 mmHg, (6) more than moderate valvular heart disease, and (7) atrial fibrillation. All enrolled patients underwent an exercise stress screening test such as a treadmill exercise test or stress myocardial perfusion scintigraphy during hospitalization, and patients with an ischemic response were excluded. We excluded patients with atrial fibrillation because the irregular rhythm effects on the speckle-tracking evaluation. The diagnosis of T2DM was based on the World Health Organization criteria [14]. The mean patient age was 61 ± 13 years, 92 (48%) patients were women, and the mean LVEF was 66% ± 5% (all  ≥  55%). For comparison, a control group including 81 age-, sex-, and LVEF-matched normal subjects were randomly chosen from our database by the observers who were not involved in echocardiographic analysis. All normal subjects did not have a history of T2DM or cardiovascular disease. This study was approved by the local ethics committee of our institution (No. B200306).

### Echocardiographic examination

All T2DM patients and normal controls underwent transthoracic echocardiography. All echocardiographic data were obtained using a commercially available echocardiographic system (Vivid E9; GE-Vingmed, Horten, Norway). Digital routine grayscale two-dimensional cine loops from three consecutive heart beats were obtained at end-expiratory apnea from standard parasternal and apical views. Sector width was optimized to allow for complete myocardial visualization while maximizing the frame rate. Standard echocardiographic measurements were obtained in accordance with the current guidelines of the American Society of Echocardiography [4].

Two-dimensional speckle-tracking strain analysis was performed for each patient using a dedicated software (EchoPAC version 113; General Electric Medical Systems) to evaluate LV longitudinal myocardial function, which was assessed in terms of GLS. In summary, apical 4-, 2-, and long-axis views were uploaded for subsequent offline GLS analysis. Longitudinal speckle-tracking strain was calculated using an automated contouring detection algorithm, and manual adjustments of the region of interest were performed, if necessary. Longitudinal strain results for the individual clips were visualized in a color-coded format and combined in a bull’s eye plot. GLS was then determined as the averaged peak longitudinal strain of 18 LV segments and was expressed as an absolute value in accordance with current guidelines [4], which also recommend expressing all strain values as absolute values, as was done in our study, to avoid confusion regarding magnitude relationships. The pre-defined cutoff for LV longitudinal myocardial dysfunction was set at GLS < 18% [4].

### Assessment of resting HR

Resting HR was determined as the average HR during echocardiography. High HR was defined as resting HR ≥ 70 beats/minute (bpm) [13, 15].

### Statistical analysis

Continuous variables are expressed as mean values with standard deviation for normally distributed data and median values with interquartile range for non-normally distributed data. Categorical variables are expressed as frequencies and percentages. The parameters of the two subgroups were compared using Student’s t-test or the Mann–Whitney U test as appropriate. Proportional differences were evaluated using Fisher’s exact test. The comparison of parameters among HR quartiles in T2DM patients and normal controls was performed using analysis of variance (ANOVA). The initial univariable logistic regression analysis to identify univariable determinants of LV longitudinal myocardial dysfunction (GLS < 18%) was followed by a multivariable logistic regression model using stepwise selection, with the P-values for entry from the model set at < 0.10. Sequential logistic models were performed to determine the incremental benefit of HR ≥ 70 bpm in relation to GLS using clinical variables including age, sex, hypertension, and HbA1c, and echocardiographic parameters including LVEF and E/e’. A statistically significant increase in the global log-likelihood χ2 of the model was considered to represent an incremental predictive value. For all steps, a P-value of < 0.05 was considered statistically significant. All analysis were performed using a commercially available software (MedCalc software version 19.6.; MedCalc Software, Mariakerke, Belgium).

## Results

### Baseline characteristics of T2DM patients and controls

The baseline clinical and echocardiographic characteristics of the 192 T2DM patients and 81 normal controls are summarized in Table 1. Clinical data showed that T2DM patients were more likely to have a higher body weight, body mass index, systolic blood pressure, heart rate, HbA1c, and prevalence of hypertension and dyslipidemia than normal controls, while echocardiographic data showed that T2DM patients were more likely to have a larger left atrial volume index, LV mass index, and E/e’ and a smaller GLS and E/A than normal controls. In addition, the comparison baseline clinical and echocardiographic characteristics of T2DM patients and normal controls according to HR quartiles are shown in Table 2 and Additional file 1.

**Table 1:** Baseline characteristics of T2DM patients and normal controls

Variables	T2DM patients (n = 192)	Normal controls (n = 81)	P value
Clinical characteristics			
Age, years	61 ± 13	57 ± 14	0.05
Gender (female), n (%)	92 (48)	44 (54)	0.34
DM duration, years	10 (2–16)	–	–
Body weight, kg	67 ± 16	59 ± 14	0.0001
Body mass index	25.4 ± 5.4	22.2 ± 3.8	< 0.0001
Systolic blood pressure, mmHg	131 ± 20	123 ± 14	0.02
Heart rate, bpm	70 ± 11	67 ± 10	0.01
eGFR, mL/min/1.73 m^2^	74.0 ± 24.0	77.8 ± 27.8	0.30
HbA1c, %	8.5 ± 2.0	5.6 ± 0.5	< 0.0001
Comorbidities, n (%)			
Hypertension	117 (61)	7 (9)	< 0.0001
Dyslipidemia	117 (61)	6 (7)	< 0.0001
Antidiabetic drugs, n (%)			
DPP-4I	97 (51)	–	–
GLP-1 RA	28 (15)	–	–
SU	42 (22)	–	–
α-GI	40 (21)	–	–
Thiazalidine	19 (10)	–	–
Metformin	95 (49)	–	–
SGLT2 inhibitor	20 (10)	–	–
Statin	80 (42)	9 (11)	< 0.0001
Calcium channel blockers	70 (36)	9 (11)	< 0.0001
β-blockers	27 (14)	6 (7)	0.12
Echocardiographic parameters			
LV end-diastolic volume, mL	69.2 ± 21.8	73.4 ± 21.2	0.14
LV end-systolic volume, mL	24.2 ± 9.9	26.0 ± 9.1	0.19
LVEF, %	66 ± 5	66 ± 5	0.70
LVMI, g/m^2^	81.4 ± 21.2	70.7 ± 19.1	0.0001
LAVI, mL/m^2^	29.5 ± 8.5	26.5 ± 8.6	0.008
E/A	0.8 ± 0.3	1.1 ± 0.3	< 0.0001
E/e’	10.9 ± 4.1	8.4 ± 2.5	< 0.0001
Tricuspid regurgitation velocity	1.4 ± 1.0	1.6 ± 1.0	0.1
GLS	17.6 ± 3.1	20.3 ± 1.9	< 0.0001

Values are mean ± SD for normally distributed data and median and interquartile range for non-normally distributed data, or n (%)

*DM *diabetes mellitus, *eGFR* estimated glomerular filtration rate, *DPP-4I* Dipeptidyl Peptidase-4 inhibitor, *GLP-1 RA* glucagon-like peptide-1 receptors agonists, *SU* Sulfonylureas, *α-GI* α-glucosidase inhibitors, *SGLT2* sodium glucose transporter type 2, *LVEF* left ventricular ejection fraction, *LVMI* left ventricular mass index, *LAVI* left atrial volume index, *E* peak early diastolic mitral flow velocity; A, peak late diastolic mitral flow velocity; e’, spectral pulsed-wave Doppler-derived early diastolic velocity from the septal mitral annulus; *GLS* global longitudinal strain

**Table 2:** Baseline characteristics of T2DM patients according to the HR quartiles

Variables	HR:44-62 bpm (n = 48)	HR:63-69 bpm (n = 48)	HR:70-77 bpm (n = 48)	HR:78-109 bpm (n = 48)	P value
Clinical characteristics					
Age, years	61 ± 14	61 ± 13	63 ± 12	58 ± 13	0.24
Gender (female), n (%)	19 (40)	23 (48)	25 (52)	25 (52)	0.58
DM duration, years	10 (8–13)	9 (7–11)	10 (7–13)	11 (8–13)	0.85
Body weight, kg	67 ± 15	66 ± 15	66 ± 17	68 ± 17	0.97
Body mass index	25.7 ± 5.7	25.9 ± 4.8	24.8 ± 6.3	25 ± 5.0	0.77
Systolic blood pressure, mmHg	129 ± 22	132 ± 19	134 ± 20	130 ± 19	0.56
Heart rate, bpm	56 ± 5	67 ± 2	74 ± 2	85 ± 7	< .0001
eGFR, mL/min/1.73 m^2^	76.4 ± 23.2	70.1 ± 24.5	74.2 ± 22.5	75.3 ± 26.1	0.6
HbA1c, %	8.8 ± 2.3	8.3 ± 1.7	9.2 ± 1.9	8.6 ± 2.0	0.2
Comorbidities, n (%)					
Hypertension	28 (58)	30 (6)	31 (65)	28 (58)	0.92
Dyslipidemia	28 (58)	25 (52)	29 (60)	35 (73)	0.15
Antidiabetic drugs, n (%)					
DPP-4I	26 (54)	17 (35)	31 (65)	23 (48)	0.04
GLP-1 RA	2 (4)	9 (19)	7 (15)	10 (21)	0.09
SU	10 (21)	8 (17)	12 (25)	12 (25)	0.7
α-GI	13 (27)	8 (17)	9 (19)	10 (21)	0.65
Thiazalidine	3 (6)	7 (15)	4 (8)	5 (10)	0.57
Metformin	20 (42)	19 (40)	30 (63)	26 (54)	0.07
SGLT2 inhibitor	4 (8)	4 (8)	6 (13)	6 (13)	0.83
Statin	20 (42)	21 (44)	18 (38)	21 (44)	0.9
Calcium channel blocker	18 (38)	21 (44)	20 (42)	11 (23)	0.16
β-blocker	5 (10)	9 (19)	6 (13)	7 (15)	0.68
Echocardiographic parameters					
LV end-diastolic volume, mL	73.4 ± 19.5	70.8 ± 19.9	70.6 ± 26.0	61.8 ± 19.8	0.05
LV end-systolic volume, mL	25.4 ± 8.5	25.3 ± 9.5	24.4 ± 11.7	21.9 ± 9.5	0.26
LVEF, %	66 ± 5	65 ± 6	66 ± 5	66 ± 5	0.82
LVMI, g/m^2^	86.8 ± 20.7	80.7 ± 19.5	82.7 ± 22.1	75.5 ± 21.4	0.08
LAVI, mL/m^2^	31.5 ± 8.0	29.3 ± 9.0	30.7 ± 8.9	26.5 ± 7.2	0.02
E/A	0.9 ± 0.3	0.8 ± 0.2	0.7 ± 0.2	0.8 ± 0.2	0.001
E/e’	11.0 ± 3.6	11.1 ± 3.5	11.6 ± 5.4	10.1 ± 3.6	0.36
Tricuspid regurgitation velocity	1.6 ± 0.9	1.5 ± 1.0	1.5 ± 1.0	0.9 ± 1.1	0.005
GLS	18.0 ± 3.0	18.0 ± 2.8	16.8 ± 2.9	17.4 ± 3.5	0.65

All abbreviations as in Table 1

### Association between HR and LV longitudinal myocardial function

**Fig. 1:**
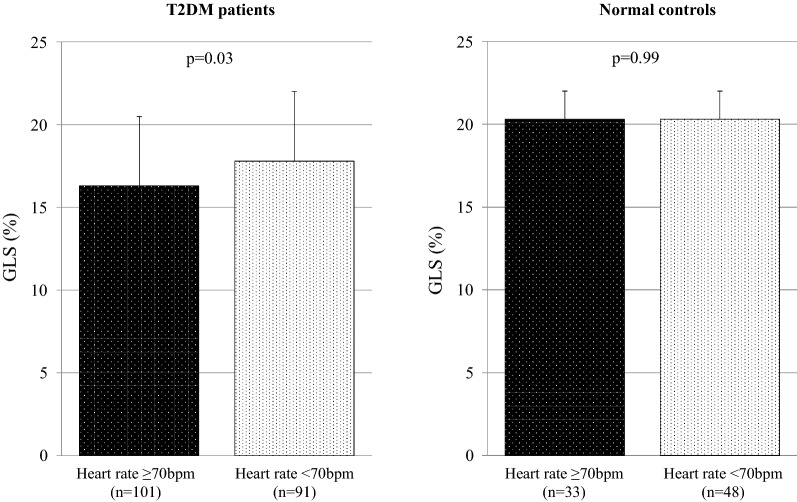
Bar graphs of GLS of T2DM patients and normal controls showing significantly higher GLS in T2DM patients with high HR than those with low HR and similar GLS between normal controls with high and low HR

Resting HR ≥ 70 bpm was observed in 101 T2DM patients, whereas it was observed in 33 normal controls. LV longitudinal myocardial function, assessed in terms of GLS, in normal controls with high and low HR was similar (20.3% ± 1.7% vs. 20.3% ± 2.0%; P = 0.99), whereas GLS in T2DM patients with high HR was significantly lower than that in T2DM patients with low HR (16.3% ± 4.2% vs. 17.8% ± 2.8%; P = 0.03; Fig. 1).

Table 3 shows the results of univariable and multivariable logistic regression analysis to identify LV longitudinal myocardial dysfunction in T2DM patients. In univariable logistic regression analysis, age, body mass index, T2DM duration, HR ≥ 70 bpm, HbA1c, E/e’, and LVEF were associated with LV longitudinal myocardial dysfunction. Multivariable logistic regression analysis showed that HR ≥ 70 bpm (odds ratio: 1.04; 95% confidence interval: 1.01–1.07; P = 0.01) was independently associated with LV longitudinal myocardial dysfunction in T2DM patients. Body mass index, T2DM duration HbA1c, LVEF, and E/e’ were also associated with LV longitudinal myocardial dysfunction in T2DM patients. Table 4 shows the results of univariable and multivariable logistic regression analysis to identify LV longitudinal myocardial dysfunction in normal controls. Unlike in T2DM patients, none of the parameters, including HR ≥ 70 bpm, were associated with LV longitudinal myocardial dysfunction in multivariable logistic regression analysis.

**Table 3:** Associated factor of GLS in T2DM patients

	Univariable			Multivariable		
	OR	95% CI	P value	OR	95% CI	P value
Age	0.97	0.95–1.00	0.02			
Body mass index	1.13	1.06–1.21	0.0002	1.14	1.07–1.23	0.0002
T2DM duration	1.0	1.0–1.01	0.05	1.0	1.0–1.01	0.01
Heart rate ≥ 70 bpm	1.03	1.00–1.05	0.04	1.04	1.01–1.07	0.01
HbA1c	1.17	1.01–1.37	0.04	1.27	1.06–1.51	0.01
LVEF	0.89	0.84–0.95	0.0003	0.86	0.8–0.93	0.0001
E/e’	1.04	1.01–1.04	0.0007	1.11	1.01–1.22	0.03
E/A	0.71	0.23–2.2	0.56			
TR velocity	0.89	0.69–1.18	0.44			
Hypertension	1.08	0.60–1.94	0.8			
Using AV node blockers	1.42	0.79–2.54	0.24			

*OR* odds ratio, *CI *confidential interval

All other abbreviations as in Table 1

**Table 4:** Associated factor of GLS in normal controls

	Univariable			Multivariable		
	OR	95% CI	P value	OR	95% CI	P value
Age	1.02	0.97 to 1.08	0.43			
Heart rate ≥ 70 bpm	0.55	0.10 to 3.05	0.50			
HbA1c	1.46	0.24 to 8.69	0.68			
LVEF	0.85	0.72 to 1.00	0.05			
LAVI	0.98	0.89 to 1.07	0.61			
E/e’	0.84	0.58 to 1.20	0.33			

*OR* odds ratio, *CI *confidential interval

All other abbreviations as in Table 1

**Fig. 2:**
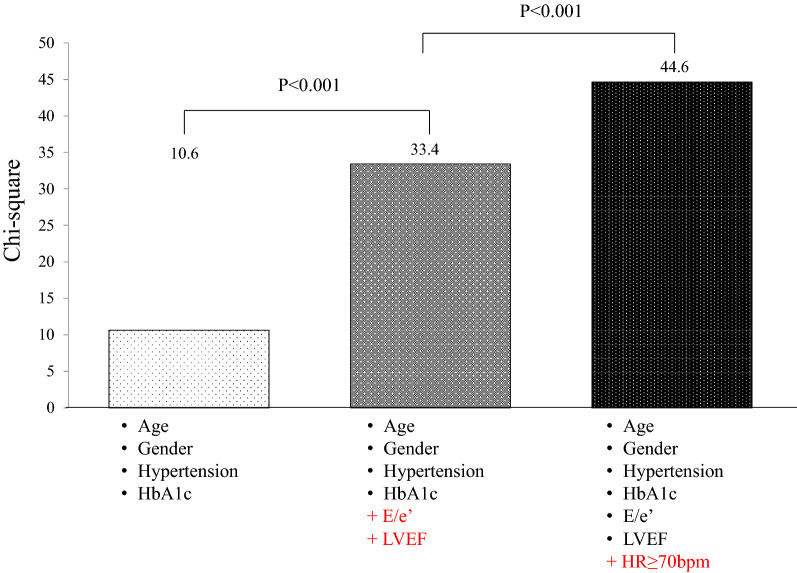
Bar graphs of sequential logistic regression models showing that one model, based on clinical variables including age, sex, hypertension, and HbA1c, showed an improvement with the addition of LVEF and E/e’ and a further improvement with the addition of HR ≥ 70 bpm

The incremental benefits determined using sequential logistic models to identify the association between GLS and clinical variables are shown in Fig. 2. One model, based on clinical variables including age, sex, hypertension, and HbA1c (χ^2^ = 10.6), showed an improvement with the addition of LVEF and E/e’ (χ^2^ = 33.4, P < 0.001) and a further improvement with the addition of HR ≥ 70 bpm (χ^2^ = 44.6, P < 0.001).

**Fig. 3:**
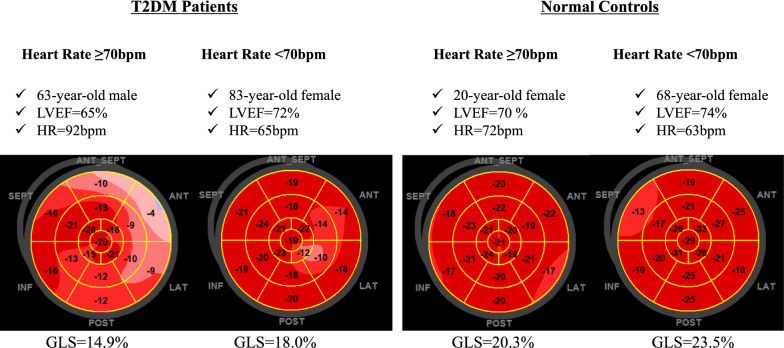
Representative cases of GLS in a bull’s eye plot of high and low HR subjects in a T2DM patient and a normal control

Figure 3 shows representative cases of GLS in a bull’s eye plot of high and low HR subjects in a T2DM patient and a normal control.

## Discussion

In our study, resting HR was associated with LV longitudinal myocardial function in T2DM patients, but such a phenomenon was not observed in age-, sex-, and LVEF-matched normal controls.

### Association between LV longitudinal function and T2DM

T2DM is a well-known risk factor for HF and is an important comorbid disease of Stage A HF, similar to hypertension. Lack of T2DM control is an important predictor of new-onset HF, with every 1% increase in HbA1c correlating to an 8%-19% increase in the incidence of HF [16, 17]. The presence of LV longitudinal myocardial dysfunction has been identified in T2DM patients with preserved LVEF without overt coronary artery disease or HF [6–12]. In addition, T2DM is a major cause of HFpEF, similar to hypertension, with HFpEF usually presenting as LV diastolic dysfunction. Some researchers have maintained that LV longitudinal myocardial dysfunction, rather than LV diastolic dysfunction, should be considered the first marker of a preclinical form of T2DM-related cardiac dysfunction in T2DM patients with preserved LVEF without overt HF [8, 18]. Ernande et al. showed that LV longitudinal dysfunction, assessed as GLS < 18%, was even present in T2DM patients with preserved LVEF and normal LV diastolic function [8]. Thus, it has been suggested that progression of uncontrolled T2DM leads to LV longitudinal myocardial dysfunction and LV diastolic dysfunction, that GLS is associated with LV diastolic function, and that reduced GLS can coexist with LV diastolic dysfunction in T2DM patients with preserved LVEF, leading to HFpEF. Therefore, assessment of LV longitudinal myocardial function is promising strategy for detection of LV myocardial damage due to T2DM, known as diabetic cardiomyopathy.

### Impact of HR on LV function in T2DM

Poanta et al. evaluated the association of HR with LV diastolic function in 58 T2DM patients without signs of cardiovascular disease [19]. They showed that HR in T2DM patients with impaired relaxation pattern of LV diastolic dysfunction was significantly higher than that in T2DM patients without LV diastolic dysfunction (91 ± 10 bpm vs. 88 ± 11 bpm, P < 0.05). Other studies focusing on the association between HR variability and LV diastolic function in T2DM patients without overt HF [19–21] have reported that HR variability parameters in T2DM patients with LV diastolic dysfunction were significantly lower than those in T2DM patients without LV diastolic dysfunction. As described above, limited studies have evaluated the association between HR with LV diastolic function in T2DM patients and the effect of HR on LV longitudinal myocardial function in asymptomatic T2DM patients with preserved LVEF is unclear. In this study, we showed that LV longitudinal myocardial function in T2DM patients with HR ≥ 70 bpm was significantly worse than that in T2DM patients with HR < 70 bpm, but this finding was not observed in normal controls. In addition, HR ≥ 70 bpm was independently associated with LV longitudinal myocardial dysfunction in T2DM patients in multivariable logistic regression analysis. LV longitudinal myocardial function in T2DM patients with preserved LVEF was already impaired compared to normal controls in this study so that T2DM patients may be susceptible to the adverse effect of high HR, leading to further early LV damage caused by high HR. However, our speculation seems to be insufficient to ascertain the existence of a true differential GLS behavior by HR between T2DM patients and normal controls. Thus, further investigation is necessary to confirm our findings.

In addition, other investigators have reported the association of exercise-induced hemodynamics evaluation with impaired HR adjustment in HFpEF patients and T2DM patients [22–25]. Borlaug et al. showed that exercise-induced elevation in pulmonary capillary wedge pressure in HFpEF patients was confirmed by greater increases in LV end-diastolic pressure and was associated with blunted increases in HR [22]. O’Connor et al. showed that a slower kinetics of adjustments of HR was more evident in older T2DM male patients with longer T2DM duration or with suboptimal glycemic control [24]. On the contrary, Caron et al. reported that well-controlled T2DM male patients and with relatively short T2DM duration did not show significant HR abnormalities with respect to control subjects [25].

### Clinical implication

High resting HR is a known marker of cardiovascular outcomes in HF patients, especially HFrEF patients and general populations [13, 15, 26]. In addition, a high HR was associated with a significantly high risk of all-cause death or cardiovascular hospitalization in HFpEF patients in sinus rhythm, similar to that observed in HFrEF patients [27]. However, it is unclear whether HR-lowering therapy is beneficial for patients with HFpEF, including those with Stage A HF. There are no large randomized controlled trials to evaluate HR lowering with β-blockers or ivabradine in HFpEF with LVEF ≥ 50% [28]. Moreover, ivabradine is currently restricted to off-label use in HFpEF patients with HR ≥ 70 bpm. Thus, we are conducting a multi-center prospective study (IVA-PEF study) to evaluate the effect of ivabradine on LV function such as LV diastolic function and LV longitudinal myocardial function in HFpEF patients, including those with Stage A HF [29]. Our findings suggest that careful observation or early therapeutic intervention with established cardioprotective medications, including ivabradine, may avoid or limit the progression of Stage A HF to Stage B HF for patients with HR ≥ 70 bpm.

## Study limitations

There were the following limitations in this study. First, this was a single-center retrospective study; hence, prospective multicenter studies with larger patient populations are needed to further assess our findings. Second, only a small number of patients were currently available for follow-up; hence, the effect of HR changes on LV longitudinal myocardial function is unclear. Third, a control group consisted of age-, sex-, and LVEF-matched normal subjects who were randomly chosen from our database, however, there were significant differences of body weight, systolic blood pressure, and the prevalence of hypertension and dyslipidemia between two groups. These parameters can effect on LV longitudinal myocardial function (GLS). Finally, high HR was defined as resting HR ≥ 70 bpm based on the previous registry for HFrEF [13, 15] in this study, but there is currently no established optimal cutoff value of high HR for predicting cardiovascular events for HFpEF patients.

## Conclusion

Compared with normal subjects, resting HR was associated with LV longitudinal myocardial function in asymptomatic T2DM patients with preserved LVEF. Our findings provide new insights on the management of T2DM patients.

## Supplementary Information

**Additional file 1:** Baseline characteristics of normal patients according to the HR quartiles.

## Data Availability

Data sharing not applicable to this article as no datasets were generated or analyzed during the current study.

## Abbreviations

Bpm: Beats/minute
E/e’: Mitral inflow E and mitral E’ annular velocities ratio
GLS: Global longitudinal strain
HF: Heart failure
HFpEF: Heart failure with preserved ejection fraction
HR: Heart rate
HFrEF: Heart failure with reduced ejection fraction
LV: Left ventricular
LVEF: Left ventricular ejection fraction
T2DM: Type 2 diabetes mellitus
