# Supplementary material for: Effect of heart rate on left ventricular longitudinal myocardial function in type 2 diabetes mellitus
Source: Cardiovasc Diabetol. 2021 Apr 24;20:87. doi: 10.1186/s12933-021-01278-7 (PMC8070299; doi:10.1186/s12933-021-01278-7)
Supplement: Supplementary file 1 — Additional file 1: Baseline characteristics of normal patients according to the HR quartiles. [file 12933_2021_1278_MOESM1_ESM.docx]

**Additional file 1**

**Baseline characteristics of normal patients according to the HR quartiles**

| Variables | HR:37-60bpm  (n=20) | HR:61-67bpm  (n=20) | HR:68-72bpm  (n=20) | HR:73-94bpm  (n=21) | P value |
| --- | --- | --- | --- | --- | --- |
| Clinical Characteristics |  |  |  |  |  |
| Age, years | 60 ± 12 | 59 ± 14 | 51 ± 13 | 59 ± 17 | 0.19 |
| Gender (female), n (%) | 9 (45) | 14 (70) | 11 (55) | 10 (48) | 0.39 |
| DM duration, years | - | - | - | - |  |
| Body weight, kg | 58 ± 12 | 58 ± 14 | 62 ± 16 | 57 ± 12 | 0.6 |
| Body mass index | 21.3 ± 2.6 | 22.9 ± 3.4 | 23.2 ± 5.2 | 21.6 ± 3.3 | 0.32 |
| Systolic blood pressure, mmHg | 126 ± 16 | 122 ± 12 | 124 ± 11 | 123 ± 18 | 0.96 |
| Heart rate, bpm | 54 ± 6 | 64 ± 2 | 70 ± 2 | 79 ± 6 | <0.001 |
| eGFR, mL/min/1.73 m^2^ | 67.8 ± 10.3 | 85.2 ± 35.6 | 68.1 ± 32.9 | 87.8 ± 21.5 | 0.06 |
| HbA1c, % | 5.7 ± 0.3 | 5.6 ± 0.7 | 5.6 ± 0.5 | 5.5 ± 0.6 | 0.83 |
| Comorbidities, n (%) |  |  |  |  |  |
| Hypertension | 1 (5) | 2 (10) | 2 (10) | 2 (10) | 0.94 |
| Dyslipidemia | 0 (0) | 1 (5) | 3 (15) | 2 (10) | 0.31 |
| Antidiabetic drugs, n (%) |  |  |  |  |  |
| DPP-4I | - | - | - | - |  |
| GLP-1 RA | - | - | - | - |  |
| SU | - | - | - | - |  |
| α-GI | - | - | - | - |  |
| Thiazalidine | - | - | - | - |  |
| Metformin | - | - | - | - |  |
| SGLT2 inhibitor | - | - | - | - |  |
| Statin | 0 (0) | 2 (10) | 4 (20) | 3 (15) | 0.23 |
| Calcium channel blockers | 0 (0) | 4 (20) | 2 (10) | 3 (15) | 0.25 |
| β-blockers | 2 (10) | 3 (15) | 1 (5) | 0 (0) | 0.3 |
| Echocardiographic Parameters |  |  |  |  |  |
| LV end-diastolic volume, mL | 82.0 ± 19.4 | 71.0 ± 20.7 | 66.6 ± 19.8 | 73.9 ± 23.0 | 0.13 |
| LV end-systolic volume, mL | 27.3 ± 8.2 | 26.1 ± 9.1 | 24 .1± 8.8 | 26.2 ± 10.5 | 0.68 |
| LVEF, % | 66 ± 5 | 67 ± 6 | 66 ± 5 | 65 ± 4 | 0.71 |
| LVMI, g/m^2^ | 71.1 ± 14.3 | 73.8 ± 17.2 | 70.2 ± 21.4 | 67.8 ± 23.2 | 0.8 |
| LAVI, mL/m^2^ | 39.4 ± 5.1 | 27.2 ± 8.0 | 24.5 ± 10.0 | 26.8 ± 10.6 | 0.68 |
| E/A | 1.1 ± 0.3 | 1.0 ± 0.4 | 1.1 ± 0.2 | 1.0 ± 0.3 | 0.59 |
| E/e’ | 9.0 ± 2.4 | 7.8 ± 2.3 | 8.2 ± 2.6 | 15.4 ± 2.7 | 0.46 |
| Tricuspid regurgitation velocity | 1.6 ± 1.0 | 1.7 ± 1.1 | 1.5 ± 1.0 | 1.7 ± 1.1 | 0.84 |
| GLS | 23.1 ± 1.9 | 20.4 ± 1.7 | 19.7 ± 1.9 | 20.3 ± 2.0 | 0.4 |

All abbreviations as in Table 1
